# Mutation du gène NOD2 chez les patients marocains atteints de la maladie de Crohn: prévalence, étude génotypique et corrélation au phénotype de la maladie

**DOI:** 10.11604/pamj.2017.27.116.9187

**Published:** 2017-06-14

**Authors:** Mouna Tamzaourte, Ikram Errabih, Hayat Krami, Fadlouallah Maha, Lahmiri Maria, Nadia Benzzoubeir, Laaziza Ouazzani, Ahmed Sefiani, Houria Ouazzani

**Affiliations:** 1 Service d’Hépato-Gastroentérologie «B», Centre Hospitalier Universitaire Ibn Sina, Rabat, Maroc; 2 Service de Génétique Médicale, Centre Nationale d’Hygiène, Rabat, Maroc

**Keywords:** MICI, maladie de Crohn, étude génétique, mutation NOD2, CARD 15, Chronic inflammatory bowel disease (IBD), Crohn’s disease, genetic study, NOD2/CARD15 mutation

## Abstract

L'objectif était de déterminer la prévalence des mutations du gène NOD2/CARD15 dans un groupe de patients Marocains atteint de Maladie de Crohn et étudier sa corrélation génotype-expression phénotypique. Etude transversale cas témoin menée sur une durée de 16 mois. Ont été inclus 101 patients atteints de la maladie de Crohn, entre Janvier 2012 et Avril 2013 ainsi qu'un groupe contrôle de 107 patients. L'analyse génétique a consisté à rechercher 3 variants du gène NOD2: p.Arg702Trp, p.Gly908Arg et p.Leu1007fsins. Puis une étude de corrélation génotype-expression phénotypique a été menée. L'analyse génétique des patients atteint de maladie de crohn a mis en évidence la présence de la mutation NOD2 chez 14 patients (13,77%) contre 7 patients (6,53%) du groupe témoin. L'étude de la fréquence des différents allèles a retrouvé la mutation de p.Gly908Arg dans 6,43%, p.Leu1007fsins dans 0,99% et p.Arg702Trp dans 0,49% contre respectivement 2,80%, 0% et 0,46% dans le groupe témoin. L'étude de la corrélation génotype, expression phénotypique a démontré que la mutation CARD15 est corrélée à une localisation iléo-caecale de la maladie, à une présentation fistulisante et sténosante ainsi qu'à une évolution sévère avec recours fréquent à la chirurgie et aux immunosuppresseurs. La prévalence de la mutation NOD2/ CARD15 dans notre série est faible. Cette mutation est corrélée à une forme grave de la maladie.

## Abstract (en)

The aim of this study was to determine the prevalence of NOD2/CARD15 gene mutations in a group of Moroccan patients with Crohn’s disease and to study its correlation with genotype-phenotypic expression. We conducted a cross-sectional case-control study over a period of 16 months. 101 patients with Crohn’s disease were enrolled between January 2012 and April 2013 as well as a control group of 107 patients. We performed a genetic analysis to identify 3 NOD2 gene variants: p.Arg702Trp, p.Gly908Arg and p.Leu1007fsins. Then we conducted a study of the correlation between genotype and phenotypic expression. The genetic analysis of patients with Crohn’s disease highlighted the presence of NOD2 mutation in 14 patients (13.77%) versus 7 patients (6.53%) in the control group. The study of the frequency of different alleles showed p.Gly908Arg mutation in 6.43%, p.Leu1007fsins in 0.99% and p.Arg702Trp in 0.49% versus 2.80%, 0% and 0.46% in the control group respectively. The study of the correlation between genotype and phenotypic expression showed that CARD15 mutation is associated with ileocecal Crohn’s disease, with fistulizing and stenosing behavior in Crohn’s disease as well as with severe evolution and frequent recourse to surgery and immunosuppressants. The prevalence of NOD2/ CARD15 mutation in our case series is low. This mutation is correlated with severe Crohn’s disease.

## Introduction

Les maladies inflammatoires chroniques de l'intestin (MICI) sont des maladies multifactorielles. Cela signifie qu'elles résultent de la combinatoire de plusieurs facteurs de risque dont certains sont génétiques et d'autres environnementaux. En d'autres termes, la maladie ne survient qu'après exposition à un (des) facteur(s) de risque environnemental (aux) sur un terrain génétiquement prédisposé. Ce terrain génétique n'est lui même pas simple à définir et consiste en une association plus ou moins complexe de plusieurs variantes génétiques à risque. La complexité des causes sous-jacentes aux MICI est inconnue et on ne sait pas aujourd'hui combien il existe de gènes de susceptibilité ni de facteurs de risque environnementaux. Les récents développements en génétique suggèrent toutefois que les gènes de susceptibilité aux MICI se comptent par dizaines ou peut être même par centaines. On ne sait pas non plus comment doivent interagir ces facteurs génétiques entre eux. Le modèle « multifactoriel » reflète donc en grande partie notre incompréhension des causes de la maladie. Cette incompréhension limite beaucoup la recherche étiologique sur les MICI. Cette dernière n'en est pas moins importante pour la compréhension de ces maladies et la mise au point de traitements spécifiques. C'est ce qui justifie l'approche génétique des MICI [1]. Plusieurs arguments sont en faveur d'une approche génétique de la maladie de Crohn (MC): l'existence d'une anamnèse familiale positive: 8 à 10% des patients atteints de MC ont un ou plusieurs patents atteints de MC [2], le taux de concordance entre jumeaux monozygotes de 20 à 62% pour la MC et 6 à 19% pour la RCH, alors que le taux de concordance pour les faux jumeaux n'est que de 0 à 6% pour la MC et 0 à 3% de RCH [3]. Le troisième argument est l'association de la MC avec d'autres syndromes génétiques [4].

Avec l'approche du clonage positionnel, il a été possible d'identifier le gène CARD15 (Caspase Recruiment Domain 15) ou NOD2 (Nucleotide oligomérisation Domain 2) situé sur le chromosome 16q12 et composé de 12 exons. Son rôle dans la MC a été validé par de nombreux auteurs [5, 6]. C'est le gène le plus fortement associé à la MC. Le NOD2 code pour une molécule intracellulaire activable par des composants de la paroi bactérienne: les muropeptides dérivés du petidoglycane. Il participe donc à la reconnaissance et à la réponse de l'hôte vis-à-vis de bactéries. Trois mutations de ce gène sont associées à une susceptibilité pour la maladie de Crohn, la R702W (arginine [R] en tryptophane [W] en position 702 de la protéine), G908R (glycine [G] en arginine [R] en position 908 de la protéine) et Leu1007fsInsC (insertion de la base cytosine en position 1007 de l'ADN codant, responsable d'un décalage de lecture (fs pour frameshift)). D'autres variantes de séquence ont été décrites (environ 700 à ce jour) mais non associés à la maladie de Crohn [7]. Dans ces études initiales, le risque relatif chez un sujet hétérozygote, pour une de ces mutations, était évalué à 3, et pour les homozygotes à 38-44. Il est important de noter qu'aucune association avec la rectocolite hémorragique n'a été démontrée. L'objectif de ce travail était de: évaluer l'implication des variants du gène NOD2 dans la susceptibilité génétique à la MC dans la population marocaine; Déterminer la prévalence des mutations du gène NOD2/CARD15 dans un groupe de patients Marocains porteurs d'une MC et étudier son expression phénotypique; Faire une corrélation entre le génotypage NOD2 et le phénotypage de la maladie de Crohn chez les malades mutés.

## Méthodes

Il s'agit d'une étude transversale cas témoin menée sur une durée d'un an conjointement entre le Service d'Hépato-Gastroentérologie « Médecine B » du Centre Hospitalier Ibn Sina de Rabat et le Service de Génétique médicale de l'Institut National d'Hygiène de Rabat. Ont été inclus tous les patients atteints de MC diagnostiquée sur des critères cliniques, endoscopiques et histologiques sur une période entre Janvier 2012 et Avril 2013. Ont été exclus tous les patients ayant une forme familiale de la MC. L'étude génétique a été réalisée après consentement oral du patient au cours de la consultation au niveau du laboratoire de Génétique Médicale. Elle a consisté après prélèvement de sang périphérique sur un tube EDTA et après extraction d'ADN, en de nombreuses techniques de biologie moléculaire pour la recherche des 3 variants du gène NOD2: p.Arg702Trp, p.Gly908Arg et p.Leu1007fsins. L'étude génétique a été réalisée également par la même technique pour un groupe contrôle de 107 patients sains. L'ADN génomique a été extrait à partir d'échantillons de sang total prélevé sur tube EDTA, en utilisant la méthode d'extraction du sel. La concentration et la qualité d'ADN ont été déterminées pour chaque échantillon. L'ADN des patients et des sujets contrôles a été amplifié pour chacun des trois variantes p.Arg702Trp, p.Gly908Arg, et p.L1007fsinsC du gène NOD2 comme indiqué précédemment. Les produits de PCR ont été séquencés en utilisant un séquenceur ABI Prism 310. Les fréquences des allèles ont été exprimées en pourcentage de chromosomes contenant un allèle variant. Toutes les analyses ont été réalisées avec le logiciel SPSS version de 17.0. Les fréquences des allèles et des génotypes chez les patients atteints de MC et chez les contrôles ont été calculées. Les associations ont été testées en comparant les fréquences des allèles de la p.Arg702Trp, p.Gly908Arg et de la p.Leu1007fsinsC chez les patients porteurs de la MC et chez les sujets contrôles, en utilisant le test exact de Fisher. Une comparaison des fréquences des génotypes entre les différents groupes a été évaluée à l'aide du test chi 2 (χ2). D'un autre côté, la comparaison entre le génotypage NOD2 et le phénotypage de la maladie de Crohn chez les malades mutés a été effectuée à l'aide du logiciel SPSS 17.0. Les variables qualitatives ont été exprimées en effectifs et pourcentages, les variables quantitatives ont été exprimées en moyenne et écart-type. Le test de Student a été utilisé pour comparer les variables quantitatives et le test de Chi2 ou le test exact de Ficher pour comparer les variables qualitatives.

## Résultats

101 patients atteints de la maladie de Crohn ont été inclus dans l'étude. Le sex ratio F/H est de 1.4. L'âge moyen des patients est de 30 ans avec des extrêmes allant de 08 à 64 ans. L'antécédent de tabagisme a été noté chez 25% des patients. 58% des patients ont une MC à localisation iléo-colique contre 30,8% à localisation colique et 11,2% iléale. Il s'agit d'une MC inflammatoire dans 80%, sténosante dans 48,6% et fistulisante dans 30,8%. Dans 52%, il s'agit d'une poussée sévère de la maladie. Les manifestations extradigestives ont été notées dans 46.3%, à type de manifestations articulaires dans 80% des cas. Et les manifestations ano-périnéales ont été notées chez 43,5% des malades. Il s'agit de lésions simples dans 78% des cas. Le recours à la chirurgie a été noté dans 48% des cas et aux immunosuppresseurs dans 49.1% des cas. La population témoin compte 107 sujets volontaires de nationalité marocaine en bonne santé apparente: 55 hommes et 52 femmes, âgés de 18 à 55 ans. Le recrutement a été effectué au cours d'un programme de don de sang au Département de génétique médicale de l'Institut national d'Hygiène à Rabat, Maroc. Ils n'avaient pas de symptômes évocateurs de maladie inflammatoire chronique de l'intestin connus ni d'antécédents familiaux de MICI. Sur les 101 patients atteints de maladie de Crohn inclus dans l'étude, l'analyse génétique a mis en évidence la présence de la mutation du gène NOD2 chez 14 patients, d'où une prévalence de 13,8% dans notre population malade. D'un autre côté, sur les 107 sujets contrôles, 07 portent une des trois mutations du gêne NOD2, d'où une prévalence chez les sujets contrôle de 6,5%. La différence entre les deux groupes n'est pas statistiquement significative (p=0,18). La fréquence des allèles des trois variantes du gène NOD2 sont présentées dans le Tableau 1. Aucune mutation combinée n'a été retrouvé chez les patients atteints de MC ou chez les sujets contrôles. La mutation p.Gly908Arg a été retrouvée dans 10.89% des cas chez les sujets malades contre 5.60% des cas des sujets contrôles. La différence entre les deux groupes n'est pas significative (p=0,21). Concernant la mutation p.Leu1007fsinsC, elle a été retrouvée dans 1.89% des sujets malades contre 0% des sujets sain (p=0,6). La troisième mutation recherchée la p.Arg702Trp, a été retrouvée à de pourcentages similaires chez les sujets malades et sains (0,99% et 0,93 respectivement, p=0,97). Le résultat des fréquences des allèles des trois variantes communes (CD p.Arg702Trp, p.Gly908Arg, et p.Leu1007fsinsC) est présenté dans le Tableau 2. La variante p.Gly908Arg était présent à une fréquence élevée dans le groupe allélique CD en comparaison avec le groupe de contrôle (p.Gly908Arg, 6,43 vs 2,8%), mais cette différence n'était pas statistiquement significative (P= 0,09). Deux patients porteurs de MC étaient porteurs hétérozygotes de la variante p.Leu1007fsinsC alors qu'aucun des témoins ne l'étaient. La fréquence allélique de cette mutation n'était pas statistiquement significative entre les deux groupes (p = 0,235). Un patient atteint de MC ainsi qu'un sujet contrôle étaient porteurs hétérozygotes pour la variante p.Arg702Trp. Les fréquences alléliques et génotypiques de p.Arg702Trp (p= 1, et p= 0,967 respectivement) n'étaient pas statistiquement différentes entre les deux groupes.

**Tableau 1:** Fréquence génotypique des différentes mutations NOD2 chez les sujets contrôlés et chez les sujets atteints de la maladie de Crohn (MC)

Mutations	Fréquence n (%)		P
	Patients atteints MC n=101	Sujets contrôlés N=107	
**pGly 908Arg**	11(10,89)	06 (5,60)	0,21
**Hétérozygote**	09	06	
**Homozygote**	02	00	
**pLeu1007fsincC**	02 (1,89)	00 (0)	0,64
**Hétérozygote**	02	00	
**Homozygote**	00	00	
**p.Arg702Trp**	01 (0,99)	01 (0,93)	0,96
**Hétérozygote**	01	01	
**Homozygote**	00	01	

**Tableau 2:** Données des principales études de la littérature ayant étudié la prévalence des différentes mutations NOD2 ainsi que la corrélation au phénotype de la maladie

***Auteurs***	***MC***	***Contrôle***	***Fréquence des allèles***			***Association démontrée avec phénotype MC***
			**R702W**	**G908R**	**1007fsinsC**	
**Abreu [** **43** **]**	201	-	8,6	5,9	5,9	Stenosant
**Ahmad [** **35** **]**	244	349	12,5	3,3	9,4	Iléon
**Brant [** **36** **]**	275	-	-	-	-	Age jeune, iléon, sténosant, fistulisant
**Cuthbert [** **39** **]**	429	290	9,1	2,0	7,0	Iléon, iléo-colique
**Esters [** **31** **]**	570	165	12,9	6,0	8,6	Colon
**Hampe [** **21** **]**	607	575	10,5	5,2	14,5	Fistulisant, iléon, colon gauche
**Helio [** **30** **]**	271	300	3,3	0,6	4,8	Iléon, fistulisant
**Mendoza [** **37** **]**	204	104	-	-	-	Fistulisant, iléon, chirurgie
**Oostengrug [** **42** **]**	369	276	9,0	4,8	9,2	Sexe féminin, âgé<40ans, iléon, anal, fistulisant, sténosant, chirurgie, histoire familiale de MICI
**Radlmayer [** **38** **]**	97	120	-	-	23,7	Sténosant, fistulisant, chirurgie
**Vavassori [** **40** **]**	133	-	-	-	-	Iléon
**Vermeire [** **41** **]**	229	71	12,9	5,2	10,3	Iléon

Par ailleurs, le séquençage direct des exons 4 et 8 du NOD2 nous a permis de détecter chez 6 patients atteints de la MC, la présence à l'état hétérozygote de la variante 2753C connue. Nous avons également détecté une autre variante inconnue: la 2105G/C, qui n'a jamais été rapportée dans les études précédentes. Elle a été retrouvée associée avec la mutation hétérozygote de la p.Gly908Arg chez un patient atteint de MC et avec la mutation p.Arg702Trp chez un autre. 08 femmes et 06 hommes sont porteurs de la mutation NOD2 dans notre série de 101 patients atteints de la maladie de Crohn. L'âge moyen est de 28ans. Trentre-trois% des patients avaient un antécédent de fissure anale et 16% un antécédent d'appendicectomie. On a noté que 28.6% des patients étaient fumeurs et que plus de la moitié des malades (57.1%) présentaient des manifestations extradigestives essentiellement à type d'arthralgies. La maladie de Crohn était de siège iléo-caecal dans 92,9% des cas et les manifestations ano-périnéales étaient associées dans 50% des cas. Se rapportant à l'histoire naturelle de la maladie de Crohn chez nos patients, une présentation inflammatoire a été notée dans 78.6%, fistulisante dans 57,1% et sténosante dans 64.3% des cas. Dans 78,6% des cas, il s'agissait d'une forme sévère de la maladie intestinale. Ces derniers ont eu recours à la chirurgie et ont bénéficié d'un traitement d'entretien à base d'immunosuppresseurs. La comparaison entre les patients porteurs de la mutation et ceux qui en sont indemnes (Tableau 3) n'a pas montré de différence statistiquement significative en matière de caractéristiques démographiques (âge moyen de début, sex ratio), de tabac et de présence de manifestations extra-digestives. Cependant, on note la fréquence quasi permanente de la localisation iléo-caecale de la MC chez les patients porteurs de la mutation par rapport à ceux qui en sont indemnes (92,9% vs 54,8%, p=0,008). 50% des malades mutés présentent des manifestations ano-périnéales. On remarque également la fréquence élevée des formes fistulisantes et sténosantes de la maladie de Crohn chez les patients NOD2+ (57,1% vs 26,9% et 64,3% vs 46,2% respectivement), mais cette différence n'est significative que pour le phénotype fistulisant (p=0,008). La comparaison entre les deux groupes a souligné la présence chez les malades mutés de formes plus sévères de la maladie de Crohn, de même qu'un recours fréquent à la chirurgie et aux immunosuppresseurs (p=0,03, p=0,01 et p=0,02 respectivement).

**Tableau 3:** Comparaison des patients atteints de la maladie de Crohn porteurs de la mutation NOD2 et ceux qui en sont indemnes

	NOD2+ n=14	NOD2- n=87	P
Sexe ratio (F/M)	1,2	1,4	NS
Age de début	28 ans	30,6 ans	NS
Tabac	28,6%	24,5%	NS
Manifestations extradigestives	57,1%	44,7%	NS
Localisation			
Iléon	0%	12,9%	NS
Colon	7,1%	32,3%	NS
**Iléo-colique**	**92,9%**	**54,8%**	**0,008**
Manifestations ano-périnéales	50%	42,6%	NS
Formes inflammatoires	78,6%	80,9%	NS
**Formes fistulisantes**	**57,1%**	**26,9%**	**0,03**
Formes sténosantes	64,3%	46,2%	NS
Sévérité			
Légère	0%	14,9%	NS
Modérée	21,4%	37,2%	NS
Sévère	78,6%	47,9%	0,03
**Recours aux immunosuppresseurs**	**78,6%**	**44,7%**	**0,01**
**Recours à la chirurgie**	**78,6%**	**44,7%**	**0,02**

## Discussion

Les maladies inflammatoires Chroniques de l'intestin, en pratique la maladie de Crohn et la rectocolite hémorragique, décrites dans la première partie du XX siècle, sont devenues ces 40 dernières années un problème de santé publique dans le monde occidental où le risque cumulé durant la vie d'avoir une MICI est de 0,5 à 1% [8]. Elles se caractérisent par une atteinte inflammatoire chronique et récidivante de la paroi intestinale chez des sujets souvent jeunes. L'hypothèse étiologique actuelle est celle des maladies multifactorielles complexes, survenant chez des individus génétiquement prédisposés, au cours desquelles une réponse immunitaire muqueuse anormale vis-à-vis de la microflore intestinale survient, déclenchée ou aggravée par des facteurs environnementaux [9, 10]. A ce jour, seuls le tabagisme et l'appendicectomie ont été reconnus comme influençant le risque d'apparition des MICI et leur évolution, même si leurs mécanismes d'action restent inconnus [11, 12]. La fréquence des formes familiales de MICI ainsi que la concordance dans 50 à 60% pour la maladie de Crohn observée chez les jumeaux homozygotes illustrent par ailleurs l'importance du terrain génétique [2, 3]. C'est en 1996, qu'un criblage large du génome humain identifia un locus possédant une liaison forte avec la maladie de Crohn et dénommé locus IBD1 situé sur le chromosome 16 (16q) [13]. En 2001, trois publications simultanées [5, 6, 14] décrivirent le gène CARD15, gène de susceptibilité pour la maladie de Crohn, et alors trois mutations ont été rapportées et impliquées dans la pathogénie de la MC.

L'implication de la mutation NOD2/ CARD 15 dans la physiopathologie de la maladie de Crohn est encore mal connue. De nombreuses études ont démontré que les maladies inflammatoires du tube digestif sont la conséquence de réponses immunitaires excessives de la muqueuse intestinale en réaction à des composants de la riche microflore et aboutissant à une activité anormale des cellules immunitaires de l'hôte. Par ailleurs, dans la MC, on observe une diminution de sécrétion des défensines de mécanisme inconnu et dont le rôle antimicrobien est bien connu. On sait aussi que l'épithélium intestinal est assez « poreux » et qu'il existe une interaction permanente entre la flore microbienne intestinale et le système immunitaire de la muqueuse. Dans les conditions normales, la flore intestinale ne provoque pas de réaction inflammatoire. En cas de déficit d'activité de NOD2/CARD15, une réaction importante de TLR2 (régulé par NOD2) aux ligands des bactéries commensales initierait au moins en partie une réaction inflammatoire [15]. Par ailleurs, des cellules spécialisées localisées à la base des cryptes de Lieberkühn dans l'intestin grêle, les cellules de Paneth, secrètent des peptides antimicrobiens dont les alphadéfensines qui sont diminuées dans la MC (ce qui n'est pas le cas des autres peptides anti-infectieux secrétés par ces cellules). Cette diminution semble liée à celle de NOD2 (par perte de fonction, conséquence des mutations) et aboutit notamment à une plus faible capacité à détruire E. coli et S. cerevisiae (phénomènes observés in vitro). Le déficit en alphadéfensine semble donc un mécanisme important dans la physiopathologie de la MC [15]. De nombreuses études épidémiologiques ont démontré la grande variation ethnique de la contribution du gène NOD2/CARD15 à la maladie de Crohn, cette contribution étant définie par la fréquence des trois mutations décrites dans ce gène. En Asie, notamment au Japon, en Corée et en Chine, dans les populations afro-américaines, CARD15 ne semble pas jouer un rôle dans le développement de la maladie de Crohn contrairement à la population européenne où une grande hétérogénéité existe entre les différents pays [16]. Ainsi, ces trois mutations représentent une susceptibilité accrue pour la maladie de Crohn avec une prévalence plus faible dans les pays du nord de l'Europe [17]. Le risque absolu de développer une maladie de Crohn chez les porteurs d'une des trois mutations est estimé à l'état homozygote à 1/25 démontrant ainsi le rôle probable d'autres facteurs environnementaux et/ou génétiques. Une méta-analyse a montré que les conséquences de chacune de ces trois mutations étaient différentes. Le risque relatif de développer une maladie de Crohn chez un patient porteur d'un allèle mutant était augmenté de 4, 3 et 2 pour respectivement Leu1007fsinsC, G908R et R702W [18]. Ce risque est multiplié par 20-40 chez les homozygotes ou hétérozygotes composites. Une mutation est retrouvée chez environ 15-20% des patients atteints de maladie de Crohn [19].

Dans notre étude, la prévalence de la mutation p.Gly908Arg était présente à une fréquence élevée dans le groupe allélique CD en comparaison avec le groupe de contrôle (p.Gly908Arg, 6,43 vs 2,8%), mais cette différence n'était statistiquement pas significative (P = 0,09). Parmi les trois variantes, le p.Gly908Arg est la moins fréquente dans la population caucasienne [5, 20-30] alors que c'est la variante majeure chez les Tunisiens [31] et dans notre population marocaine, avec association non significative. Concernant la prévalence de la variante p.Leu1007fsinsC, elle est portée de façon hétérozygote par deux patients marocains atteints de MC alors qu'aucun des témoins ne l'étaient. La fréquence allélique de cette mutation n'était pas statistiquement différente (p = 0,235) entre les deux groupes. Ces résultats sont semblables à ceux retrouvés dans la population tunisienne [31], bien qu'une susceptibilité à la maladie de Crohn pour cette variante a été rapportée pour certaines populations caucasiennes [20, 21]. Un patient marocain et un contrôle étaient hétérozygotes pour la variante p.Arg702Trp. Les fréquences alléliques et génotypiques de p.Arg702Trp (p= 1, p=0,967) n'étaient pas statistiquement différentes entre les deux groupes. La fréquence de cette variante dans la population marocaine est inférieure à celles rapportées chez les Tunisiens [32] et les Caucasiens [20, 21]. Nous pouvons conclure suivant notre étude que le gène NOD2 n'est pas associé avec la MC dans la population marocaine, comme il a déjà été démontré chez nos voisins tunisiens [32]. L'association de la MC avec les trois principales variantes du gène NOD2 a été trouvée chez les Australiens [22], les Américains [6], Les Européens [5], et les populations caucasiennes américaines. Une association significative entre la mutation de la variante p.Gly908Arg et p.Leu1007fsinsC et avec la MC a été également démontré chez les juifs ashkénazes, en Hollande, en Finlande, en Irlande et en Ecosse [23, 24]. Par contre, la mutation des trois variantes n'a pas été retrouvée ni chez les patients atteints de MC ni chez les témoins dans les populations asiatiques [33, 34]. Cette variabilité des fréquences alléliques dans les différents groupes ethniques confirme la complexité de l'origine de la maladie de Crohn et donne des preuves de la variabilité génétique du gène NOD2/CARD15 dans les différentes populations. Après les études de prévalence, de nombreuses études se sont intéressées à la corrélation génotype / phénotype de la maladie de Crohn. A ce jour, plus de 10 études de corrélation au phénotype ont été publiées [7, 22, 30, 31, 35-41] (Tableau 2) ainsi qu'une méta-analyse [42] publiée en 2006 par une équipe hollandaise incluant 21 études portant sur une population essentiellement caucasienne. L'atteinte iléale est l'association qui a été retrouvé de façon constante dans la plupart de ces études. Egalement démontrés dans les études précédentes, la forte association de la mutation NOD2/CARD15 avec l'âge jeune de début, la localisation iléo-colique et une maladie de Crohn compliquée (phénotype sténosant, fistulisant et recours accru à la chirurgie) [21, 22, 30, 36-38, 43].

Egalement rapportée par 2 études (une espagnole et une étude canadienne) mais non étudiée dans notre cohorte, l'association entre la mutation CARD 15 et l'antécédent familial de maladies chroniques inflammatoires de l'intestin [37, 39]. En effet, ces études ont démontré l'association des trois mutations du gène NOD2 avec la présence d'une histoire familiale de MICI (p=0.08 pour chaque mutation). Ce qui tend encore une fois en faveur de l'approche génétique de la maladie de Crohn. Le sexe féminin a également été retrouvé par Lesage [7] et Oostenbrug [42] comme associée à la mutation NOD2. Cette association n'a pas été mise en évidence dans la méta-analyse [42] (p=0.8 et p=0.3 pour 1007fsinsC et pour les 2 autres mutations respectivement) de même que par notre étude qui n'a pas mis en évidence d'association entre la mutation NOD2 et le sexe. Les phénotypes associés avec la mutation du NOD2 qui ont été retrouvés dans la plupart des études et aussi dans la méta analyse [42], sont la localisation iléale de la maladie retrouvée avec les 3 mutations mais plus fortement avec 1007fsingC, l'âge jeune de diagnostic retrouvé uniquement chez les patients porteurs de plus d'une mutation, le phénotype de la maladie (sténosant et fistulisant) ainsi que le recours accru à la chirurgie retrouvés essentiellement avec les mutations G908R et 1007fsingC. Dans notre étude, l'association avec l'atteinte iléale n'a pas été mis en évidence, c'est plutôt la localisation iléo-colique qui semble être corrélée à la mutation NOD2 (92,8% vs 54,8% p=0,008). Concernant l'âge de début de la maladie, on n'a pas retrouvé de différence significative entre les malades mutés et les malades non mutés (28 ans vs 30.6 ans). Seule la forme fistulisante de la maladie a été retrouvée corrélée avec la mutation NOD2 (p=0,03), alors qu'il n'y a pas de différence statistiquement significative concernant la forme sténosante de la maladie de Crohn (64% vs 46% p= NS). 78,6% des patients mutés présentent une forme sévère de la maladie contre 47,9% (p= 0,03) cependant cette association n'a pas été retrouvée dans la littérature. Contrairement à l'association avec le recours à la chirurgie (78,6% vs 44,7% p=0,01) qui a été largement retrouvée dans la littérature.

## Conclusion

La prévalence de la mutation NOD2/ CARD15 dans notre série reste faible et non associée à la maladie de Crohn. Cette mutation est par contre corrélée à une localisation iléo-caecale, une présentation sténosante et fistulisante et un recours accru à la chirurgie et aux immunosuppresseurs.

### Etat des connaissances actuelle sur le sujet

- Le gène NOD2/CARD 15 est le plus fortement associé a la maladie de Crohn;
- L'atteinte iléale, l'âge jeune de début ainsi qu'une présentation plus grave de la maladie sont souvent associés à la mutation de ce gène.

### Contribution de notre étude à la connaissance

- La prévalence de la mutation NOD2/ CARD15 au Maroc reste faible et non associée à la maladie de Crohn;
- Cette mutation est par contre corrélée à une forme plus grave de la maladie.

## Conflits d’intérêts

Les auteurs ne déclarent aucun conflit d'intérêts.
